# A systems framework for national assessment of climate risks to infrastructure

**DOI:** 10.1098/rsta.2017.0298

**Published:** 2018-04-30

**Authors:** Richard J. Dawson, David Thompson, Daniel Johns, Ruth Wood, Geoff Darch, Lee Chapman, Paul N. Hughes, Geoff V. R. Watson, Kevin Paulson, Sarah Bell, Simon N. Gosling, William Powrie, Jim W. Hall

**Affiliations:** 1 School of Engineering, Newcastle University, Newcastle upon Tyne NE1 7RU, UK; 2 Committee on Climate Change, 7 Holbein Place, London SW1 W 8NR, UK; 3 School of Mechanical, Aerospace and Civil Engineering, The University of Manchester, Manchester M13 9PL, UK; 4 Anglian Water, Lancaster House, Lancaster Way, Ermine Business Park, Huntingdon, Cambridgeshire PE29 6XU, UK; 5 School of Geography, Earth and Environmental Sciences, University of Birmingham, Edgbaston, Birmingham B15 2TT, UK; 6 Department of Engineering, Durham University, Durham DH1 3LE, UK; 7 Faculty of Engineering and the Environment, University of Southampton, University Road, Southampton SO17 1BJ, UK; 8 School of Engineering and Computer Science, University of Hull, Cottingham Rd, Hull HU6 7RX, UK; 9 Institute for Environmental Design and Engineering, UCL, 14 Upper Woburn Place, London WC1H 0NN, UK; 10 School of Geography, University of Nottingham, Nottingham NG7 2RD, UK; 11 Environmental Change Institute, University of Oxford, South Parks Road, Oxford OX1 3QY, UK

**Keywords:** infrastructure, climate change, risk assessment, interdependence, systems approach

## Abstract

Extreme weather causes substantial adverse socio-economic impacts by damaging and disrupting the infrastructure services that underpin modern society. Globally, $2.5tn a year is spent on infrastructure which is typically designed to last decades, over which period projected changes in the climate will modify infrastructure performance. A systems approach has been developed to assess risks across all infrastructure sectors to guide national policy making and adaptation investment. The method analyses diverse evidence of climate risks and adaptation actions, to assess the urgency and extent of adaptation required. Application to the UK shows that despite recent adaptation efforts, risks to infrastructure outweigh opportunities. Flooding is the greatest risk to all infrastructure sectors: even if the Paris Agreement to limit global warming to 2°C is achieved, the number of users reliant on electricity infrastructure at risk of flooding would double, while a 4°C rise could triple UK flood damage. Other risks are significant, for example 5% and 20% of river catchments would be unable to meet water demand with 2°C and 4°C global warming respectively. Increased interdependence between infrastructure systems, especially from energy and information and communication technology (ICT), are amplifying risks, but adaptation action is limited by lack of clear responsibilities. A programme to build national capability is urgently required to improve infrastructure risk assessment.

This article is part of the theme issue ‘Advances in risk assessment for climate change adaptation policy’.

## 1. Introduction

Infrastructure provides the critical services such as heating, lighting, mobility and sanitation that are essential for modern society. The current variability in climate already compromises infrastructure performance, and disruption or complete failure of these services causes significant adverse social, economic and environmental impacts. For example, inundation of water treatment plants and electricity distribution substations left hundreds of thousands of people without power and water in southwest England [1]. In 2012, Hurricane Sandy caused substantial damage to New York's infrastructure networks; however, loss of the services these networks provided magnified other economic losses and also hampered recovery efforts [2]. During the winter of 2013/14, storms in the UK led to loss of power for over 150 000 homes for significant periods of time, closure of Gatwick Airport, disruption of rail/road travel including complete severance of the South Devon Main Line in Devon for two months, in addition to general damage to buildings and to other infrastructure assets [3]. A year later, more floods disrupted electricity supplies for tens of thousands of people, caused the failure of a number of bridges, and disrupted mobile and broadband communication networks [4]. The importance of infrastructure and the significant impacts from its disruption are echoed in other extreme weather events around the world [5,6,7].

Climate change will alter average weather conditions and the nature of extreme weather in the UK and globally [8]. Gradual shifts in long-term trends (e.g. a rise in average temperatures) will reduce the capacity and efficiency of some infrastructure. This will be compounded by increases in the frequency of severe weather events, such as flooding, which will lead to increased disruption of infrastructure. Climate change can thereby alter the design life of infrastructure and the effectiveness of the services it provides. Globally, $2.5tn a year is currently spent on infrastructure [9]. In the UK alone, the National Infrastructure Plan [10] sets out £300 billion of planned investment across all sectors of infrastructure by 2020/21. Infrastructure is typically associated with large capital costs and with lifespans of 30–200 years. Furthermore, there is limited flexibility once built. Overall, given the sensitivity of infrastructure performance to climate and that decisions on design and renovation have long-lasting implications which are hard to reverse, assessing the climate risks to infrastructure must therefore be a priority. To avoid longer term impacts on people and the economy, it is essential that future infrastructure investments, as well as the adaptation of existing infrastructure, are made in the context of these risks.

National assessments typically consider a broad range of climate change impacts (table 1), including water, transport and energy infrastructure sectors, but usually have only limited or no consideration of climate risks to solid waste, information and communication technology (ICT), flood and coastal protection infrastructure. Almost all the national assessments studied assess risks on the basis of published evidence, and while this is summarized in different ways, very few assessments prioritize and rank the risks identified, even in relative terms. In the UK, the 2008 Climate Change Act requires a national climate change risk assessment every 5 years. As a result, the first UK Climate Change Risk Assessment [11] was completed in 2012 and took the approach of constructing a series of quantitative response functions that related climate variables (e.g. sea-level rise) to risk (e.g. coastal flood risk) across a number of sectors. The approach developed here, and subsequently applied in the second UK Climate Change Risk Assessment [12], does not seek to replicate the detailed analysis of the first, but instead uses it as a starting point for a systematic review and assessment of new evidence of current and future risks to infrastructure. The methodology is introduced in §2, and its application to assess risks to UK infrastructure reported in §3. The paper concludes with discussion about the key findings, efficacy of the approach especially in comparison to the first UK assessment, and uncertainties and recommendations for future development of methods for national assessment of climate change risks to infrastructure.

**Table 1.** Summary of the infrastructure aspects in past national climate change risk assessments. A tick indicates that risks to the infrastructure associated with the sector were considered; for example, in all assessments flood risks were considered but the focus was on the impact of flooding rather than the flood and coastal erosion infrastructure. A cross indicates that the infrastructure sector was not assessed systematically or in detail, although in some cases risks to the sector were acknowledged in a brief statement.

		infrastructure sector						
national assessment	summary of approach	water	flood and coastal erosion	energy	transport	ICT	solid waste	interdependencies
UK 2012 [11]	— quantitative risk assessment — sector specific response function created that relate magnitude of impacts to climate change hazard — limited sub-national (Wales, Scotland, Northern Ireland) assessment	✓	×	✓	✓	×	×	— water for energy generation — recognition, but limited analysis, of a number of other modes of infrastructure interdependence
UK 2017 [12]	— review of published evidence — limited sub-national (England, Wales, Scotland, Northern Ireland) assessment — assessment of the urgency of adaptation action for each risk	✓	✓	✓	✓	✓	✓	— water for energy generation — transport for resource movements — cascading impacts of energy disruption — smartening of infrastructure with ICT — geographical co-location
USA 2014 [13]	— review of published evidence — sub-national assessment of 8 regions and the coastal zone	✓	×	✓	✓	×	×	— water for energy generation, and some other water-energy interactions — cascading impacts of energy disruption — recognition, but limited analysis, of a number of modes of interdependencies in urban and rural areas
Canada [14]	— review of published evidence — limited sub-national (Northern, Coastal regions, Great Lakes) assessment — limited analysis of relative importance of risks	✓	×	✓	✓	×	×	— water for energy generation — recognition, but limited analysis, of a number of other modes of infrastructure interdependence
Netherlands 2015 [15]	— review of published evidence — broad in coverage but not as in-depth analysis of literature — limited analysis of relative importance of risks	✓	×	✓	✓	✓	×	— water for energy generation — transport for resource movements — cascading impacts of energy disruption — smartening of infrastructure with ICT — international dependencies
Finland [16]	— synthesis of findings from a number of national scale research programmes — limited analysis of relative importance of risks	✓	×	✓	✓	×	×	— recognition, but limited analysis, of a number of modes of infrastructure interdependence
Australia [17]	— review of published evidence — limited analysis of relative importance of risks	✓	×	✓	✓	×	×	— recognition, but limited analysis, of cascading failures and water for energy generation
South Africa [18]	— review of published evidence — broad coverage of risks but limited analysis of relative importance — no sub-national assessment	✓	×	✓	✓	×	×	— interdependencies between water infrastructure, food and biofuel. — other non-infrastructure interdependencies
Germany [19]	— review of published evidence	✓	×	×	✓	×	×	— no explicit consideration of energy infrastructure, but interdependencies between water infrastructure, food and biofuel considered — other non-infrastructure interdependencies

## 2. A framework for climate risk assessment of national infrastructure

Climate change risk assessment of infrastructure needs to consider a wide range of current and possible future climatic conditions, their related risks and opportunities for infrastructure sectors, and the extent to which current or planned policies and proposals will manage them.

### (a) A systems view of infrastructure

An infrastructure risk assessment must consider more than just impacts to physical components and assets such as tracks, pipes and wires. It is crucial to consider the resources that these physical components move about, and the services they provide that the public and businesses depend upon. Furthermore, these systems are all interconnected. Increasingly, infrastructure depends on other infrastructure to work, not just technically, but also socially and economically.

**Figure 1.**
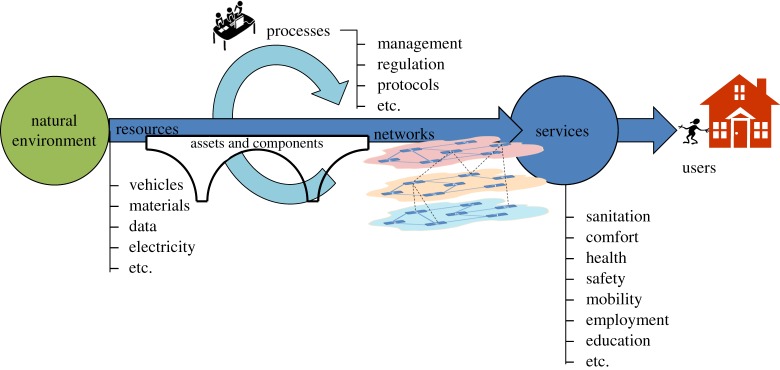
Conceptual view of infrastructure as a system (from [20]). (Online version in colour.)

A risk assessment must therefore take a systems view of infrastructure that requires consideration of a number of key elements beyond just the obvious physical assets (figure 1). Infrastructure plays an important role in modulating both the use of *natural environment* resources that is directly affected by climate change, for example water resources, but also for mitigating environmental hazards, such as hydrological extremes, that perpetrate climate risks. Individual *physical assets* interconnect to provide a *network* that joins locations demanding a particular resource or service, with areas that can supply it. The *resources* conveyed by infrastructure include vehicles, water, electricity and data as well as the materials used in infrastructure construction which enable *services* such as warmth, mobility, sanitation, transportation, and communication that benefit a wide range of individual, business, or other *users*. These are all influenced by an array of actors, institutions, regulation, protocols and *processes* that have influence over all parts of the infrastructure system. Climate change can impact directly the different constituent elements of the infrastructure system, while actions taken to manage climate risks may be implemented across any of these elements.

### (b) Systems risk assessment of infrastructure

**Figure 2.**
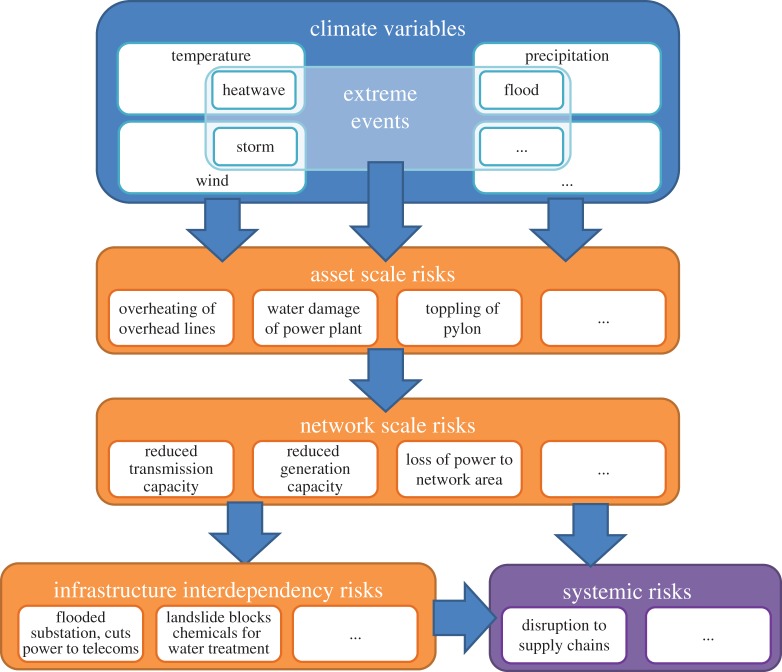
A systems approach to a climate change risk assessment framework for infrastructure, with some indicative variables or risks at each assessment stage (from [20]). (Online version in colour.)

A systems approach to climate change risk assessment of infrastructure has therefore been developed that comprises a number of stages (figure 2).

(i) Analysis of climate variables (e.g. rainfall, temperature and wind) to understand change over time, and how the frequency and magnitude of hazards such as floods or heat waves will subsequently be altered. The climate change context used in the 2017 UK Climate Change Risk Assessment is summarized in [21,22].
(ii) Characterization of each infrastructure asset, in particular its fragility and capacity, to understand its response to extreme events and changes in climate. Typically, climate loadings of larger magnitude or wider spatial coverage increase the likelihood of failure or lead to greater reduction of performance of individual assets, and consequently the impacts of failure.
(iii) Analysis of network-wide effects that occur as a result of impacts on individual or multiple components and system functions. Typically, higher climate loadings, and events that directly impact more of the network, lead to increased impacts. However, the magnitude of impacts is also mediated by network properties such as the number of backup or redundant components.
(iv) Analysis of interactions and interdependencies between infrastructure networks to understand cascading impacts.
(v) Assessment of systemic risks that are related to the loss of infrastructure services that consequently lead to indirect impacts on economic growth, social wellbeing and environmental protection. These broader interactions are considered in [23].
(vi) Adaptations may be implemented across the infrastructure system. This may involve asset- or network-scale engineering, policy or regulatory interventions, or working with users to manage demand for services.

The significance of a risk depends on the combination of the likelihood of a climatic event and its impacts. The magnitude of impacts is often mediated by asset characteristics, including fragility, capacity and redundancy. They are further mediated by the capacity and vulnerability of organizations and users affected. Low likelihood, high impact events require different management to more frequent, low impact events. In particular, the lowest probability events require special attention in terms of warning and community preparedness as it may not be possible to identify the hazards, let alone protect against them. A climate change risk assessment should therefore consider a full range of loadings, impacts and possible responses.

Infrastructure adaptation options can be compared on the basis of the impact that they are expected to have on reducing the frequency and severity of climate effects. There are four main strategies to manage climate change risks to infrastructure.

(i) Reduce the likelihood of infrastructure component failure by providing enhanced protection.
(ii) Improve the performance of infrastructure components so they are able to operate under a wider range of climatic conditions.
(iii) Provide redundancy to increase the capacity, number of alternative connections and diversity of components and backup systems.
(iv) Build capacity in organizations and communities, and via technological advancement, to deliver a fast and effective response to, and recovery from, climate disruption.

In a systems assessment, climate change adaptation is not limited to ‘major’ engineering options, but a wider set of interventions across the whole infrastructure system at a range of temporal and spatial scales. Adaptations include technical options but also regulatory, policy and community responses are crucial to enhancing the adaptive capacity (potential to adapt to climate variability and change) of infrastructure systems. However, much of the evidence of adaptation activity for UK infrastructure focuses on engineering responses, as the benefits of these are typically easier to assess quantitatively.

### (c) Evidence assessment

**Figure 3.**
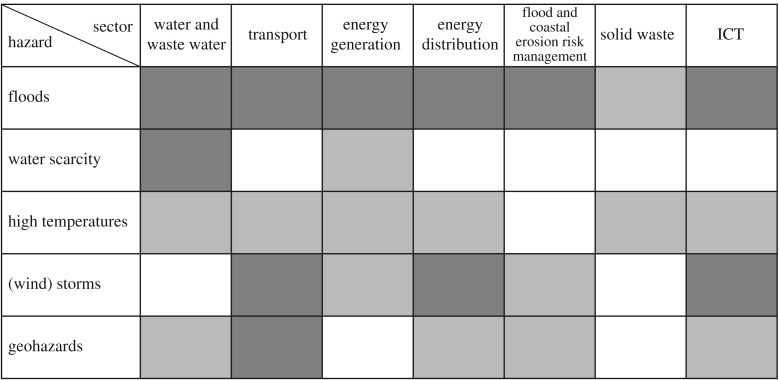
Key relationships between climate hazards and each infrastructure sector. A light shade denotes a relationship exists, a darker shade denotes the relationship is strong. Dependencies between infrastructures are not shown here.

The first stage of the risk assessment is to identify where there are causal relationships between infrastructure sectors and individual climate hazards (figure 3), which might be altered as a result of climate change.

Models and quantitative evidence exist for some of the aspects of the systems risk assessment framework in figure 2, but information on climate risks to infrastructure is recorded in a variety of formats and in a wide range of papers, reports and other material. Furthermore, the quality of the evidence base is extremely variable across the many infrastructure sectors, individual assets and their interdependencies. In the absence of individual models, or even national scale models for all the individual sectors, available evidence was analysed using the framework set out by Warren [21] to answer three questions:

1. What is the current and future level of climate risk to infrastructure?
2. To what extent are these risks going to be managed, and what is the subsequent residual risk, taking into account adaptation commitments and autonomous adaptation?
3. Are there benefits of further action in the next 5 years?

In each case the quality of the evidence was considered to determine the level of uncertainty (low, medium or high confidence) in the assessment. Subsequently, and taking into account reported adaptation actions, an assessment was conducted about the urgency and type of adaptation action required over the next 5 years to manage these long-term risks.

In all, 309 sources of evidence [20] were reviewed and used to identify priority risks in the present day and under future climatic and socio-economic conditions. An initial assessment was made by the authors, before submitting it to two external reviews by stakeholders from over 30 academic, government and non-government organizations, as well as infrastructure utility companies and consultancies. Over 650 comments were received as part of this review process which led to several refinements as assumptions and expert judgements incorporated new evidence [24]. An assessment of the urgency of climate change risks to infrastructure is summarized in table 2. Key contributions from the evidence base are summarized in the following sections, and the rationale is described in full by the Committee on Climate Change [25].

**Table 2.** Summary of the adaptation urgency for climate change risks to UK infrastructure. The adaptation urgency applies to the entire United Kingdom (England, Wales, Scotland and Northern Ireland) unless specified.

more adaptation action needed over next 5 years above those already planned	sustaining current adaptation action is sufficient to manage risks	research needed to enable assessment of the need for action	maintain a watching brief of monitoring and review of needs
— risks to infrastructure services from river, surface water and groundwater flooding — risks to public water supplies from drought and low river flows (England & Wales) — risks to infrastructure services from coastal flooding and erosion (England & Wales) — risks of sewer flooding due to heavy rainfall — risks to transport networks from embankment failure — risks of cascading failures from interdependent infrastructure networks	— risks to public water supplies from drought and low river flows (Scotland & Northern Ireland) — risks to transport, ICT and energy infrastructure from extreme heat — opportunities for water, transport, digital and energy infrastructure from reduced frequency of extreme cold events	— risks to bridges and pipelines from high river flows and bank erosion — risks to infrastructure services from coastal flooding and erosion (Scotland & Northern Ireland) — risks to energy, transport and ICT infrastructure from high winds and lightning — risks to offshore infrastructure from storms and high waves (England, Scotland & Wales)	— risks to hydroelectric generation from low or high river flows — risks to subterranean and surface infrastructure from subsidence — risks to electricity generation from drought and low river flows — risks to offshore infrastructure from storms and high waves (Northern Ireland)

## 3. Climate change risks to the UK's infrastructure

**Figure 4.**
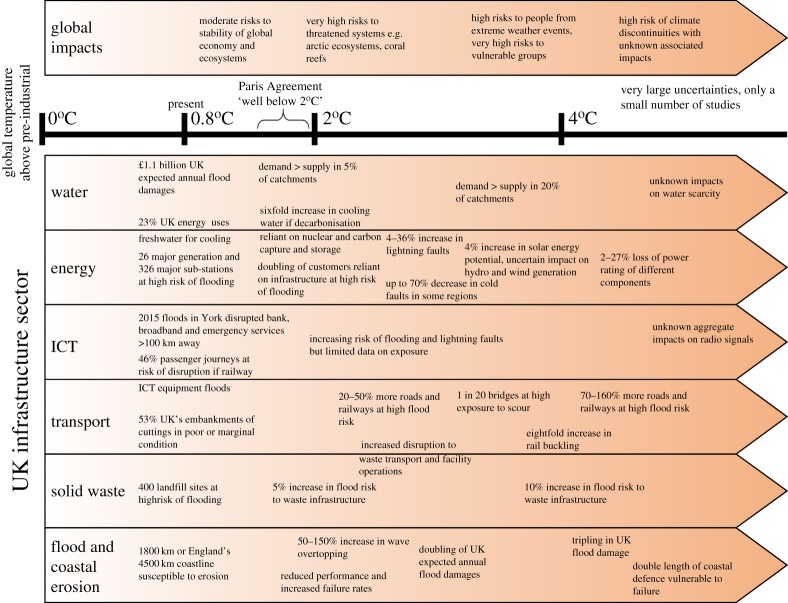
Relationship between global temperature change relative to pre-industrial era, and some of the key climate change risks to UK infrastructure. A high risk of flooding has a likelihood of flooding more frequently than 1 in 75 years (AEP ≥ 0.013). The assessment of global change risks is taken from [12]. (Online version in colour.)

Each infrastructure sector faces a number of climate risks, the magnitude of which is modulated by specific geographical or engineering features. The most significant risks for UK infrastructure are identified and summarized according to climate risk in the following sections, and by sector in figure 4. The main opportunity identified is a reduced frequency, although not cessation, of extreme cold events that impact upon water, transport, digital and energy infrastructure. However, reduced frequency of severe events can increase vulnerability to individual extreme events if the capacity to cope is reduced, as occurred during the particularly cold Winter 2010/11 [26].

### (a) Flood damage and disruption

Flooding is the most significant climate change risk to UK infrastructure, affecting all infrastructure sectors. A significant number of infrastructure assets are already situated in locations that are exposed to river, coastal, groundwater or surface water flooding. Flooding of infrastructure can lead to lengthy disruption and high repair costs. Table 3 shows that a significant proportion of infrastructure has been found to be at risk of flooding from multiple sources [27].

**Table 3.** Percentage of some infrastructure assets at risk from different sources of flooding.

	source of flooding		
	river or coastal	surface water	groundwater
power stations	41	6	18
railway track	17	9	17
railway stations	14	3	16
motorways and A-roads	9	6	9
clean water and wastewater treatment plants	33	12	24

Flood risk from river, coastal, pluvial and groundwater sources is projected to increase across the UK, even after accounting for the most ambitious adaptation plans by national and local authorities. Under a scenario of 4°C of global warming by the 2080s, the number of assets exposed could double. For example, currently some 2400 km of the UK rail network is vulnerable to flooding and this could rise by 120% by the 2080s [27]. More intense rainfall associated with this scenario will also increase the frequency of sewer flooding and combined sewer overflow events. Infrastructure networks near rivers will be at risk from projected higher flows and subsequent river bank erosion. Bridges are especially vulnerable: historically the annual probability of observing a flood event in which one or more railway bridges fails is 1 in 2.6 years (annual exceedance probability, AEP = 0.390) [28]. Projected changes to winter river flows would increase scour by over 8% at 1 in 20 of all the 4239 railway and 8664 main road bridges, placing them at high risk of failure by 2080 [29].

Coastal infrastructure is particularly at risk from storm surges and rising sea levels, as well as increased rates of coastal erosion in some locations. Rising sea levels of 0.5–1 m by the end of the century will increase the proportion of assets vulnerable to coastal flooding, as well as increasing rates of coastal erosion in some locations. The annual cost of maintenance of coastal defence could increase by 150–400% [30].

### (b) Droughts and reduced water availability

The UK currently has an overall surplus of water availability, of approximately 2000 ml/day. However, supply and demand are finely balanced in many catchments. In the absence of further adaptations, by the 2050s, a high population growth and high climate change scenarios will see widespread deficits which will be largest in south-east England and the conjunctive use zones in the north of England [31]. Extended periods of low rainfall, and associated low river flows and groundwater levels, will reduce the availability of water resources, both for consumption but also for freshwater abstractions to cool power plants.

### (c) Storm damage and disruption

Overhead cables used for energy distribution, electrified rail and some ICT networks such as those delivering broadband to rural areas are vulnerable to lightning strikes, high winds and tree- and debris-related damage associated with storms. There is broad uncertainty surrounding climate projections for wind, but lightning strike disruptions to the energy network may increase between 4–36% within different regions by the 2080s under a 3°C climate scenario [32], with a similar increase in the incidence of damage to mobile phone base stations. The impact of such events is relatively low, compared to events such as flooding, as damage can usually be repaired comparatively quickly and services rapidly restored.

### (d) Geohazards (including subsidence and landslides)

Extended periods of rainfall increase slope and embankment instability. This risk is most significant for road and rail infrastructure, where nearly 2% of the UK's network is at high risk of landslide disruption and a further 6% at medium risk [29]. On average, 50 landslides per year disrupt rail services, although over the winter of 2013/14 there were 105 earthwork failures on the rail network alone [33]. The site-specific characteristics of slopes and embankments makes it especially difficult to take a proactive approach to adaptation.

Subsidence due to shrink–swell processes, and desiccation-cracking with associated reduction in stability, is driven by cycles of drought and heavy rain [34]. This can damage railway track, road surfaces and buried infrastructure such as waste and water pipes. Risks are most significant in areas where shrink–swell susceptible clay soils dominate, especially around London and the east of England. The impact of climate change on persistent rainfall events is also uncertain, but recent prolonged periods of rainfall have acted as triggers for geohazards, for example the M3 motorway was closed for two days during the 2013/14 winter storms following the sudden appearance of a sinkhole [35].

### (e) High temperatures

Railways, ICT and electricity generation, transmission and distribution infrastructure are all susceptible to extremes in temperature. The 2003 heat wave cost £2.5 million in repairs to the rail network, and the frequency of rail buckling events is expected to be four times higher under a 2°C climate change scenario [36]. Track can be pre-tensioned to suit prevailing temperatures but the greater range of high and low temperatures likely to be experienced over a year may cause operational difficulties.

Increases in air and water temperatures affect the output and efficiency of steam and gas turbine-based generators. This would also decrease the effective capacity of electricity networks by reducing the average rating of overhead lines in the distribution network by 6–10% by the 2080s for the 4°C climate scenario, although the reduction could be up to 27% for some components [37,38]. However, reductions in performance are smaller than recent historical load growth which has typically been 1.5–2% per annum [37].

### (f) Infrastructure interdependencies

Infrastructures are increasingly dependent on each other—for power, control (via ICT) and access for deliveries or servicing. Moreover, a range of other mechanisms can create interdependencies that impact upon climate risks [39]. All UK infrastructure sectors have identified failure of another infrastructure sector as a risk to their own networks [40]. However, despite efforts in recent years to encourage infrastructure operators to work together and address vulnerabilities [41], there is usually insufficient information to fully appraise the risks between systems. There is presently no formalized framework for engagement and collaborative working which, when coupled with commercial and security sensitivities, remain barriers to routine data sharing and cooperation. In this assessment, five dependency risks were identified as especially sensitive to climate change risks.

#### (i) Dependence on water infrastructure

The UK's current national energy generation mix requires significant volumes of water for cooling, and 23% of the UK's energy is generated by power plants cooled from freshwater sources [42]. Inland power generation in England is most vulnerable to reduced water availability [43]. Water demand for cooling is influenced by the electricity generation mix, decarbonization strategies that involve high levels of carbon capture and storage could double freshwater consumption by the 2050s [44]. Even with no climate change impacts the projected growth of cooling water abstractions could reach the current licensed abstraction limit in some catchments by the 2040s [45]. This would compound the drought risk described previously which did not consider electricity generation mix.

#### (ii) Dependence on power infrastructure

All infrastructure sectors require power for some, if not all, of their assets. Analysis of the impact of a 1 in 1000 year flood (AEP = 0.001) in the Thames catchment showed approximately 12 million people rely on power infrastructure within the floodplain [46]. Moreover, loss of this power infrastructure could disrupt wastewater treatment infrastructure serving over 7 million people, water supply infrastructure serving over 10 million and telecommunications infrastructure serving over 9 million. Although this analysis did not have information on the level of flood protection of individual assets, it highlights the importance of electricity infrastructure in supporting other sectors. Current trends in energy infrastructure, such as uptake of electric vehicles and the electrification of railway lines, and decentralization of energy systems, will alter the nature of these risks although there is limited evidence of the long-term implications.

#### (iii) Dependence on information and communication technology infrastructure

Modern infrastructure is increasingly reliant on ICT for monitoring, remote operation, clock synchronization, and coordination of emergency response during extreme events. For example, the loss of power to an Internet hub in Italy led to failure of other power stations disrupting 56 million people across Italy and Switzerland [47]. There has been no comparable event in the UK, but during the 2015/16 winter floods in York damaged assets supporting wired and wireless ICT networks, causing loss of banking, broadband and emergency services at distances of up to 100 km away [48]. Insufficient data about the location of ICT assets and their role in managing other infrastructure sectors has hindered comprehensive analysis. However, ICT has been shown to be the second most important infrastructure network for the operation of the UK's rail network; for example, flooding of the 7% of assets in the low flood risk (less than 1 in 200 year likelihood of flooding) would disrupt 46% of passenger journeys [49]. Although there is limited available data about assets and adaptation actions, it is known that ICT is increasingly important to the operation of all other infrastructure networks, and it has been shown that this increased interdependency would also increase the risk of cascading failure across the entire infrastructure system and beyond [47,50].

#### (iv) Dependence on transport infrastructure

Infrastructure networks are often dependent on transport infrastructure for continued operation, for example to ensure access for resources such as fuel, personnel and emergency response. Failure of key infrastructure components such as bridges, or landslides that block important transport corridors, can significantly increase travel times as a result of rerouting of journeys [51]. Loss of the only road bridge in Workington in 2009 required residents to make a two-hour journey to reach the other side of the river causing significant local social and economic impacts [52]. A 1 in 200 year (AEP = 0.005) flood event in Newcastle-upon-Tyne would block multiple roads simultaneously, and during peak travel time this would cause disruption equivalent to 1000 passenger days [53]. Analysis of a 1 in 200 year flood event on key fuel and food depots in the Shetland Islands, shows that the subsequent disruption to supply chains could lead to depletion of stocks across the region within a few days, echoing observations in New York after hurricane Sandy [54].

#### (v) Geographical dependence

Co-sited cables, fibre optics, road, railway, pipe and other infrastructure—even if not physically connected but running in parallel along the same route—can amplify climate risks as a single event can disrupt multiple services unless assets are designed to interoperate [55]. Limited information on these risks exists, but using best available data hotspots of geographical infrastructure interdependence across England and Wales have been identified [56]. These hotspots reflect the number of users directly or indirectly dependent on all infrastructure in any given location. Unsurprisingly, large population centres are shown to be concentrations of interdependencies, but less intuitively many hotspots are in the urban periphery areas as critical assets are usually not located in city centres, while critical infrastructure ‘corridors’ that span the country are also revealed, highlighting the risk to disruption of multiple services from a large scale event [56].

## 4. Discussion

### (a) Balancing detail and scope in national risk assessment

This analysis, undertaken as part of the second UK Climate Change Risk Assessment, had a wider scope than other national assessments (table 1) as it included a broader analysis of risks to other sectors such as solid waste, ICT, flood and coastal erosion infrastructure. Furthermore, it considered all infrastructures together, including assessment of a number of risks resulting from infrastructure interdependence. In the 5 years since the first [11] assessment there has been improved analysis of risks from flooding, bridge scour, rail buckling, windstorms and interdependencies. Although the results are not comparable in absolute terms, no manifest change in the trends of key risks was identified, and the risks still outweigh the opportunities.

The methodology used in this national scale assessment allows integration of evidence in a consistent way, is more appropriate for handling uncertainties, and can incorporate qualitative evidence that is ill-suited to more quantitative modelling exercises. However, by only relying on, and interpreting, existing evidence there is limited opportunity for learning from new analysis; although it does afford the opportunity to identify knowledge gaps where extra analysis is required. The classification of urgency used here was tailored to support development of a National Adaptation Plan mandated by the UK Climate Change Act; however, the principle of translating risk information into a recommendation for action is useful as it provides a direct link between the assessment and policy requirements.

### (b) Consistency of climate risk management information

This analysis reviewed a large body of evidence, which revealed enormous diversity in methodologies, quality and completeness of information analysed. Studies have used a wide range of different scenarios, spatial scales, timeframes and impact metrics to assess risks. The majority of evidence is compiled from observations or sub-national analyses, with only a small proportion of the evidence providing a national-scale assessment of infrastructure risks. However, many national scale studies are reliant on the same underpinning datasets (e.g. the Environment Agency's NaFRA data layer provides a national flood depth-probability map [57]). Furthermore, evidence is not evenly distributed across the range of climate risks to infrastructure. While there is an abundance of material on flood risks, and to a lesser extent on drought risks, a comprehensive national assessment of slope stability risk currently defies analysis because of the sensitivity of asset performance to very specific local conditions.

Across the UK's infrastructure sectors there are a wide range of approaches to infrastructure governance, regulation, data collection and data accessibility. Some infrastructures like roads are in the public sector (though sometimes privately operated) while others, like telecoms and England's water industry, are in the private sector; for railways a hybrid ownership approach exists. This poses challenges for risk assessment, but especially when considering the risk reduction benefits of adaptation because measures are reported inconsistently, and in many cases not recorded at all. This lack of standardization and incomplete availability of data has posed a significant challenge for this assessment, and highlights a current lack of a systematic approach to understanding climate risks on infrastructure. There are good reasons to encourage diversity in the data and methods to build the evidence basis for climate change risk assessment, this can ensure a broader set of insights, but avoids too much correlation and overlap in the evidence base. However, a shared and open approach to recording the metadata associated with key attributes of the evidence (e.g. geography; timeframes; scenarios; recording adaptation measures) is strongly recommended as this would greatly facilitate the assimilation of risk information.

### (c) Significant knowledge gaps

A number of significant uncertainties, beyond consistency of reporting, and gaps in our understanding of infrastructure risks have been identified that should be addressed by more fundamental research.

The impact of climate change on wind, lightning, offshore waves and currents, and sub-hourly rainfall intensity, is highly uncertain. However, there is a direct relationship between these weather processes and infrastructure risks, especially for assets designed for a long lifespan. Developments in high-resolution modelling of convective storms [58] may be able to reduce the uncertainties related to these risks.

Infrastructure performance, including deterioration over the long-term and incidents of failure, is poorly recorded. Given the long lifespan of many assets and the timeframes over which climate change manifests, this is a significant uncertainty in risk assessment. More comprehensive laboratory and long-term field research, as exemplified by [59], are required to improve understanding of long-term infrastructure deterioration and failure processes. This must be complemented by a more exhaustive, consistent, and forensic approach by infrastructure stakeholders to recording and analysing the limited sample of infrastructure failures.

Interconnections and dependencies between infrastructure systems are already important. The impact of cascading failures across infrastructures has shown that it can be enormous, but even for smaller scale events these interactions can compound impacts. Furthermore, infrastructure is increasingly reliant on international connections, whether physical asset connections between countries, or movement of resources to operate infrastructure services. Methods to enable these risks to be analysed are emerging [39,60] but in-depth understanding of these risks and the modes by which failure is transmitted, remain poor and requires greater study. This knowledge gap is greatest for those interdependencies related to ICT, where analysis is hampered by limited knowledge of the location of assets and the logical operation of these systems. As infrastructure becomes increasingly interdependent, with ICT having the most critical role in the ‘smartening’ of infrastructure and cities, it is increasingly important to understand these risks.

## 5. Conclusion

Infrastructure merits specific consideration in climate change risk assessment, and due to increased interdependency, the multiple infrastructure sectors warrant collective consideration. A systems framework for national scale climate change risk assessment for infrastructure has therefore been developed and used to analyse over 300 sources of data to prioritize adaptation actions for the UK's infrastructure. The assessment shows that infrastructure in the UK is already experiencing significant impacts as a result of the natural variability of our climate. Projected changes in climate will reduce the life expectancy of existing infrastructure and the effectiveness of the services it provides. These risks far outweigh potential opportunities, such as reduced cold-related disruption, associated with climate change. Furthermore, climate change will interact with, and exacerbate, the impact of other pressures that include population growth and ageing infrastructure.

There is evidence that significant adaptation steps to manage climate change risks have been implemented, or are underway, across most infrastructure sectors. Where sufficient information is available to assess their effectiveness, these adaptation investments will maintain, or in some instances reduce, climate risks over the next decade or two. However, beyond this projected changes in climate are likely to outpace current adaptation plans.

While understanding of risks to individual infrastructure sectors has improved, the impacts of climate change are expected to be amplified by interconnectivities and interdependencies between these sectors. Understanding of these is less comprehensive, and current governance arrangements mean that responsibilities for assessing and managing risks from interdependencies are unclear. This remains an area of priority for future research.

The method presented here for assessing climate change risks to national infrastructure is appropriate given the limited availability, and substantial variation, of evidence and tools to support a national scale assessment. However, given the importance of infrastructure to the functioning of a modern society, there is a need to enhance capabilities in infrastructure climate change risk assessment. A starting point will be agreement of a common baseline, some standardized socio-economic and adaptation scenarios to provide common reference points (but not limit development of other scenarios), and improved records and metadata about adaptation actions. However, to fully tackle the issues described in the discussion, a national capability needs to go further and must ultimately provide a common and internally coherent analytical framework that enables different risks to be fairly compared. It must be able to analyse the impact of ‘persistent’ events (e.g. repeated sequence of storms or floods, in the same or multiple locations) and simultaneous hazards (e.g. wind storm coupled with flooding). This can only be achieved by producing a national database of the location, function, design and condition of assets, and a record of any adaptation to these assets in order to provide a reliable assessment of current and future infrastructure performance. This work has highlighted that there is a substantial body of work that can be built upon, however, research and development is currently disjointed and hampered by a lack of sustained investment.

## Data accessibility

Evidence and information compiled as part of the 2017 UK Climate Change Risk Assessment can be accessed here: www.theccc.org.uk/tackling-climate-change/preparing-for-climate-change/uk-climate-change-risk-assessment-2017/

The full reference list of material incorporated into the infrastructure section of the 2017 UK Climate Change Risk Assessment is provided in that chapter:

Dawson RJ *et al.* 2016 Infrastructure. In *UK Climate Change Risk Assessment Evidence Report*, chapter 4. Report prepared for the Adaptation Sub-Committee of the Committee on Climate Change, London.

## References

[1] Pitt M 2008 The Pitt review: learning lessons from the 2007 floods. London, UK: Cabinet Office.

[2] City of New York. 2014 *PlaNYC: A stronger, more resilient New York*. New York, USA. See http://www.nyc.gov/html/sirr/html/report/report.shtml.

[3] Environment Agency (2016). https://www.gov.uk/government/publications/the-costs-and-impacts-of-the-winter-2013-to-2014-floods.

[4] Marsh T, Kirby C, Muchan K, Barker L, Henderson E, Hannaford J 2016 *The winter floods of 2015/2016 in the UK - a review*. Wallingford, UK: NERC/Centre for Ecology & Hydrology. See http://nora.nerc.ac.uk/515303/

[5] Chang SE, McDaniels TL, Mikawoz J, Peterson K 2007 Infrastructure failure interdependencies in extreme events: power outage consequences in the 1998 Ice Storm. Nat. Hazards 41, 337–358. (10.1007/s11069-006-9039-4)

[6] Ziervogel G, New M, Archer van Garderen E, Midgley G, Taylor A, Hamann R, Stuart-Hill S, Myers J, Warburton M 2014 Climate change impacts and adaptation in South Africa. Wiley Interdiscip. Rev. Clim. Change 5, 605–620. (10.1002/wcc.295)

[7] McEvoy D, Ahmed I, Mullett J 2012 The impact of the 2009 heat wave on Melbourne's critical infrastructure. Local Environ. 17, 783–796. (10.1080/13549839.2012.678320)

[8] IPCC 2013 *Climate Change 2013: the physical science basis. Contribution of Working Group I to the Fifth Assessment Report of the Intergovernmental Panel on Climate Change* (eds TF Stocker). Cambridge, UK: Cambridge University Press.

[9] Woetzel J, Garemo N, Mischke J, Hjerpe M, Palter R 2016 Bridging global infrastructure gaps. San Francisco, CA: McKinsey Global Institute.

[10] Treasury HM 2015 National infrastructure plan 2014. London, UK: HM Treasury.

[11] HR Wallingford, AMEC Environment & Infrastructure UK Ltd, The Met Office, Collingwood Environmental Planning, Alexander Ballard Ltd, Paul Watkiss Associates & Metroeconomica. 2012 *The UK Climate Change Risk Assessment 2012 Evidence Report: Project deliverable number D.4.2.1, Release 8*. London, UK: Department for Environment Food and Rural Affairs. See https://www.gov.uk/government/publications/uk-climate-change-risk-assessment-government-report.

[12] Committee on Climate Change (2016). https://www.theccc.org.uk/tackling-climate-change/preparing-for-climate-change/uk-climate-change-risk-assessment-2017/.

[13] US Global Change Research Program (2014). http://nca2014.globalchange.gov/.

[14] Warren FJ, Lemmen DS (eds). 2014 *Canada in a changing climate: sector perspectives on impacts and adaptation*. Ottawa, ON: Government of Canada. See http://www.nrcan.gc.ca/environment/resources/publications/impacts-adaptation/reports/assessments/2014/16309.

[15] Vonk M, Bouwman A, van Dorland R, Eerens H 2015 *Worldwide climate effects: risks and opportunities for the Netherlands*. The Hague, The Netherlands: PBL Netherlands Environmental Assessment Agency. See http://www.pbl.nl/en/publications/worldwide-climate-effects-risks-and-opportunities-for-the-netherlands.

[16] Ministry of Agriculture and Forestry. 2014 *Finland's National Climate Change Adaptation Plan 2022*. Helsinki, Finland: Ministry of Agriculture and Forestry. See https://ilmasto-opas.fi/en/etusivu.

[17] Government of Australia. 2015 *National Climate Resilience and Adaptation Strategy*. Canberra, Australia: Government of Australia. See http://www.environment.gov.au/climate-change/adaptation/strategy.

[18] Department of Environmental Affairs. 2016 *Climate change trends, risks, impacts and vulnerabilities: South Africa's 1st Annual Climate Change Report*. Pretoria, South Africa: Department of Environmental Affairs. See https://www.environment.gov.za/otherdocuments/reports/monitoring_climatechange_responses.

[19] Zebisch M, Grothmann T, Schröter D, Hasse C, Fritsch U, Cramer W 2005 *Climate change in Germany: vulnerability and adaptation of climate sensitive sectors*. Dessau, Germany: Federal Environmental Agency. See http://www.umweltbundesamt.de/sites/default/files/medien/publikation/long/2974.pdf.

[20] Dawson RJ (2016).

[21] Warren RF, Wilby RL, Brown K, Watkiss P, Betts RA, Murphy JM, Lowe JA 2018 Advancing national climate change risk assessment to deliver national adaptation plans. Phil. Trans. R. Soc. A 376, 20170295 (10.1098/rsta.2017.0295) 29712791 PMC5938631

[22] Humphrey K, Murphy J 2016 Introduction. In UK Climate Change Risk Assessment Evidence Report, Chapter 1. Report prepared for the Adaptation Sub-Committee of the Committee on Climate Change, London. https://www.theccc.org.uk/wp-content/uploads/2016/07/UK-CCRA-2017-Chapter-1-Introduction.pdf.

[23] Street R et al. 2016 Cross-cutting issues. In UK Climate Change Risk Assessment Evidence Report: chapter 8. Report prepared for the Adaptation Sub-Committee of the Committee on Climate Change, London. https://www.theccc.org.uk/wp-content/uploads/2016/07/UK-CCRA-2017-Chapter-8-Cross-cutting-issues.pdf.

[24] Committee on Climate Change. 2016 *Chapter review comments*. London, UK: Committee on Climate Change. See https://www.theccc.org.uk/wp-content/uploads/2016/07/CCRA-Chapter-Review-Comments-locked.xls.

[25] Committee on Climate Change. 2016 *UK Climate Change Risk Assessment 2017: Synthesis Report appendix: Urgency scoring tables*. London, UK: Committee on Climate Change. See https://www.theccc.org.uk/wp-content/uploads/2016/07/UK-CCRA-2017-Synthesis-Report-Appendix.pdf.

[26] Andersson AK, Chapman L 2011 The impact of climate change on winter road maintenance and traffic accidents in West Midlands, UK. Accid. Anal. Prev. 43, 284–289. (10.1016/j.aap.2010.08.025) 21094326

[27] Sayers PB, Horritt M, Penning-Rowsell E, McKenzie A 2015 *Climate Change Risk Assessment 2017: projections of future flood risk in the UK*. Research undertaken by Sayers and Partners on behalf of the Committee on Climate Change. London, UK: Committee on Climate Change. See https://www.theccc.org.uk/publication/sayers-for-the-asc-projections-of-future-flood-risk-in-the-uk/.

[28] van Leeuwen Z, Lamb R 2014 Flood and scour related failure incidents at railway assets between 1846 and 2013. Skipton, UK: JBA Trust. See http://www.jbatrust.org/wp-content/uploads/2016/01/JBA-Trust-Flood-and-scour-failure-at-railway-assets-1846-to-2013-W13-4224-FINAL.pdf.

[29] HR Wallingford (2014). https://www.theccc.org.uk/wp-content/uploads/2014/07/5-MCR5195-RT003-R05-00.pdf.

[30] Sutherland J, Wolf J 2002 *Coastal defence vulnerability 2075*. Report SR 590. London, UK: Department for Environment, Food and Rural Affairs.

[31] HR Wallingford, Centre for Ecology and Hydrology, British Geological Survey, Amec Foster Wheeler (2015).

[32] McColl L, Palin E, Thornton H, Sexton D, Betts R, Mylne K 2012 Assessing the potential impact of climate change on the UK's electricity network. Clim. Change 115, 821–835. (10.1007/s10584-012-0469-6)

[33] Adaptation Sub-Committee (2014). http://www.theccc.org.uk/wp-content/uploads/2014/07/Final_ASC-2014_web-version-4.pdf.

[34] Glendinning S, Hughes PN, Helm P, Chambers J, Mendes J, Gunn D, Wilkinson P, Uhlemann S 2014 Construction, management and maintenance of embankments used for road and rail infrastructure: implications of weather induced pore water pressures. Acta Geotech. 9, 799–816. (10.1007/s11440-014-0324-1)

[35] Muchan K, Lewis M, Hannaford J, Parry S 2015 The winter storms of 2013/2014 in the UK: hydrological responses and impacts. Weather 70, 55–61. (10.1002/wea.2469)

[36] Jenkins K, Glenis V, Ford A, Hall J 2012 A probabilistic risk-based approach to addressing impacts of climate change on cities: the Tyndall Centre's Urban Integrated Assessment Framework. UGEC Viewp. 8, 8–11. See https://ugec.org/docs/ugec/viewpoints/ugec-viewpoints-8.pdf.

[37] Energy Networks Association 2011 Electricity Networks Climate Change Adaptation Report. London, UK: ENA.

[38] Hu X. 2015 Impacts of Climate Change on Power Systems, PhD Thesis, University of Manchester, UK See http://ethos.bl.uk/OrderDetails.do?uin=uk.bl.ethos.679994

[39] Dawson RJ 2015 Handling interdependencies in climate change risk assessment. Climate 3, 1079–1096. (10.3390/cli3041079)

[40] DEFRA (2013). https://www.gov.uk/government/publications/adapting-to-climate-change-2013-strategy-for-exercising-the-adaptation-reporting-power.

[41] Cabinet Office (2016). https://www.gov.uk/government/publications/sector-security-resilience-plan-2016.

[42] Environment Agency (2013).

[43] Naughton M, Darton RC, Fung F 2012 Could climate change limit water availability for coal-fired electricity generation with carbon capture and storage? A UK case study. Energy Environ. 23, 265–282. (10.1260/0958-305X.23.2-3.265)

[44] Byers E, Hall J, Amezaga J 2014 Electricity generation and cooling water use: UK Pathways to 2050. Glob. Environ. Change 25, 16–30. (10.1016/j.gloenvcha.2014.01.005)

[45] Byers EA, Hall JW, Amezaga JM, O'Donnell GM, Leathard A 2016 Hydroclimatic impacts on thermoelectric power with carbon capture and storage. Environ. Res. Lett. 11, 024011 (10.1088/1748-9326/11/2/024011)

[46] Pant R, Thacker S, Hall JW, Alderson D, Barr S In press Critical infrastructure impact assessment due to flood exposure. J. Flood Risk Manag. 11, 22–33. (10.1111/jfr3.12288)

[47] Buldyrev S, Parshani R, Paul G, Stanley H, Havlin S 2010 Catastrophic cascade of failures in interdependent networks. Nature 464, 1025–1028. (10.1038/nature08932) 20393559

[48] Hill L 2016 Flooding in York cuts off North East phone lines to police and hospitals. *Chronicle*, 28 December. See http://www.chroniclelive.co.uk/news/north-east-news/flooding-york-cuts-north-east-10658579.

[49] Pant R, Hall JW, Blainey SP 2016 Vulnerability assessment framework for interdependent critical infrastructures: case-study for Great Britain's railway network. Eur. J. Transp. Infrastruct. Res. 16, 174–194.

[50] Fu G, Dawson RJ, Khoury M, Bullock S 2014 Interdependent networks: vulnerability analysis and strategies to limit cascading failure. Eur. Phys. J. B 87, 148 (10.1140/epjb/e2014-40876-y)

[51] Postance B, Hillier J, Dijkstra T, Dixon N 2017 Extending natural hazard impacts: an assessment of landslide disruptions on a national road transportation network. Environ. Res. Lett. 12, 014010 (10.1088/1748-9326/aa5555)

[52] Affleck A, Gibbon J 2016 Workington: a case study in coordination and communication. Proc. Inst. Civ. Eng-Munic. Eng. 169, 109–117. (10.1680/jmuen.15.00004)

[53] Pregnolato M, Ford A, Robson C, Glenis V, Barr S, Dawson RJ 2016 Assessing urban strategies for reducing the impacts of extreme weather on infrastructure networks. R. Soc. open sci. 3, 160023 (10.1098/rsos.160023) 27293781 PMC4892443

[54] Brown S, Dawson RJ 2013 Disruption of resource movements from flooding. In Urban flood resilience (eds Butler D, Chen AS, Djordjevic S, Hammond MJ), pp. 125–126. Exeter, UK: Centre for Water Systems, University of Exeter.

[55] Khoury M, Bullock S, Fu G, Dawson RJ 2015 Improving measures of topological robustness in networks of networks and suggestion of a novel way to counter both failure propagation and isolation. J. Infrastruct. Complex. 2, 1–20. (10.1186/s40551-015-0004-9)

[56] Thacker S, Barr S, Pant R, Hall J, Alderson D 2017 Geographic hotspots of critical national infrastructure. Risk Anal. 37, 2490–2505. (10.1111/risa.12840) 28605055

[57] Environment Agency. 2015 *NaFRA Spatial Flood Likelihood Category Grid*. See https://data.gov.uk/dataset/risk-of-flooding-from-rivers-and-sea1.

[58] Kendon EJ, Roberts NM, Fowler HJ, Roberts MJ, Chan SC, Senior CA 2014 Heavier summer downpours with climate change revealed by weather forecast resolution model. Nat. Clim. Change 4, 570–576. (10.1038/nclimate2258)

[59] Hughes PN, Glendinning S, Mendes J, Parkin G, Toll DG, Gallipoli D, Miller P 2009 Full-scale testing to assess climate effects on embankments. Eng. Sust. Proc. Inst. Civ. Eng. 162, 67–79. (10.1680/ensu.2009.162.2.67)

[60] Hall JW, Tran M, Hickford AJ, Nicholls RJ 2016 The future of national infrastructure: a system-of-systems approach. Cambridge, UK: Cambridge University Press.

